# Association between prenatal maternal anxiety and/or stress and offspring's cognitive functioning: A meta‐analysis

**DOI:** 10.1111/cdev.13885

**Published:** 2022-12-29

**Authors:** Garance Delagneau, E. Sabrina Twilhaar, Renee Testa, Sarit van Veen, Peter Anderson

**Affiliations:** ^1^ Turner Institute for Brain and Mental Health, School of Psychological Sciences Monash University Clayton Victoria Australia; ^2^ Obstetrical Perinatal and Pediatric Epidemiology Research Team Institute of Health and Medical Research Centre of Research in Epidemiology and Statistics Université Paris Cité Paris France; ^3^ Murdoch Children's Research Institute Royal Children's Hospital (Dept of Mental Health) Parkville Victoria Australia

## Abstract

This meta‐analysis examined the relationship between prenatal maternal stress and/or anxiety and the outcomes of children aged 3 months to 9 years. Of the 8754 studies published before June 2021 that were synthesized, 17 conducted in Western countries were included in the meta‐analysis (*N*_total_ = 23,307; *M*_males_ 54%; *M*_ethnicity_ White 77%, Pacific 15%, African American/Black 10%, Middle Eastern 7%, Eastern 8%). Effect sizes ranged from −0.41 to 0.15. A weak negative association was found between prenatal stress and/or anxiety exposure and children's general intellectual development. Associations varied based on the type of exposure. Findings are limited to developed counties and cannot be generalized to low‐ and middle‐income countries. Directions for maternal prenatal intervention and future studies are discussed.

**Abbreviation**
SIGN: Scottish Intercollegiate Guideline Network appraisal tool

Many women worldwide experience some levels of stress during pregnancy. Studies report that the prevalence of maternal stress during pregnancy typically ranges from 5.5% to 78% and increased considerably during the COVID‐19 pandemic (Boekhorst et al., 2021; Lynn et al., 2011; Pope et al., 2021; Vijayaselvi et al., 2015; Woods et al., 2010; Zilver et al., 2021). There is now reliable evidence that maternal prenatal stress is associated with negative health and neurobehavioral outcomes in the offspring, such as delayed cognitive development in infants and toddlers (Bussières et al., 2015; Lautarescu et al., 2020; Madigan et al., 2018; Manzari et al., 2019; Van den Bergh et al., 2020). Whether these associations extend to older children and specific cognitive domains (e.g., attention and memory), however, remains uncertain. Furthermore, our understanding of the relationship between maternal prenatal stress and children's cognitive development is hampered by the lack of consistency in the way stress is defined and measured across studies and differences in the way children's cognitive outcomes are examined (Nast et al., 2013). Therefore, a quantitative review is needed on the association between prenatal stress and children's cognitive development which (1) separates the concepts of prenatal stress, (2) examines a range of cognitive outcomes beyond early childhood, and (3) separates different types of cognitive assessment.

Stress has previously been defined and measured as exposure to stressors, perceived stress, or stress response (Epel et al., 2018). Exposure to stressors refers to the presence of stimuli, such as life events, environmental factors, and daily hassles, which cause emotional difficulty (Hahn & Smith, 1999; Killen et al., 2013). Perceived stress is an emotional reaction characterized by feelings or thoughts that arise from individuals' appraisal of life stressors and vary based on several factors, including one's social support, personality traits, and coping behaviors (Epel et al., 2018; Lazarus & Folkman, 1984; Nast et al., 2013). A stress response is most commonly characterized as biological changes, including changes in cortisol levels, reduced uteroplacental blood flow in pregnant women, increased activation of the sympathetic nervous system, decreased activation of the parasympathetic nervous system, and increased secretion of catecholamines (Chu et al., 2021; De Weerth & Buitelaar, 2005). It is triggered by an event that is perceived to be stressful and mediated by factors such as personality traits and cognitive appraisal processes (Gaab et al., 2005; Thau & Sharma, 2020). Interestingly, past studies have found a reduced physiological stress response in pregnant women, including lower cardiovascular stress reactivity and catecholamine response, which may protect the developing fetus from exposure to low levels of stress during pregnancy (De Weerth & Buitelaar, 2005). Nonetheless, physiological stress responses in pregnant women remain present, highlighting the need to further explore these mechanisms and their impact on the developing fetus.

Stress has often been linked with anxiety. This is because clinical signs of anxiety resemble those of stress (e.g., faster breathing, faster heartbeat; Graeff & Zangrossi Junior, 2010; Tsigos & Chrousos, 2002). Stress and anxiety are also underpinned by similar biological reactions (e.g., activation of the hypothalamic–pituitary–adrenal axis and sympathetic nervous system; Graeff & Zangrossi Junior, 2010; Tsigos & Chrousos, 2002). Furthermore, anxiety can constitute a source of stress as well as a reaction to it (Selye et al., 1982; Siegman, 1993). Therefore, past studies have often considered anxiety as a psychological response to stress. Given the frequent inclusion of anxiety as part of a psychological reaction to stress, this review will focus on both concepts. Another related construct is depression. Stress is often recognized as a vulnerability factor for depression, however, depression has distinct clinical symptoms and pathophysiology and has been differentiated from stress in past studies (Brigitta, 2002). For these reasons, as well as a recent meta‐analysis demonstrating an association between prenatal depressive symptoms and child development (Faleschini et al., 2019), we have not focused on maternal depression in this review.

Anxiety is a multi‐dimensional construct, comprising state, and trait anxiety. State anxiety refers to situation‐specific and transient negative emotion characterized by psychological and physiological reactions, such as irritability, difficulties concentrating, palpitations, and a reduction in serotonin release (Briley & File, 1991; National Institute of Mental Health, N. I. H., 2018; Vytal et al., 2012). Trait anxiety refers to an enduring characteristic of a person which predisposes them to experience more intense degrees of fear and worry across many situations that most people would not find threatening (Endler & Kocovski, 2001). Pregnancy‐related anxiety has also been described, which refers to worries specifically related to pregnancy, such as giving birth or bearing a handicapped child (Bayrampour et al., 2016).

While related, exposure to stressors, perceived stress, stress response, trait anxiety, state anxiety, and pregnancy‐related anxiety constitute unique psychological constructs with distinct measurement methods, duration, causes, and outcomes (Endler & Kocovski, 2001; Epel et al., 2018; Nast et al., 2013; Saviola et al., 2020; Sullivan et al., 2000). Studies have shown that different aspects of stress and types of anxiety have independent associations with health and birth outcomes (Endler & Kocovski, 2001; Epel et al., 2018; Schetter & Tanner, 2012; Wadhwa et al., 1993). As such, exposure to these different constructs may have a differential impact on the cognitive development of the child. Supporting this premise, meta‐analytic findings of Tarabulsy et al. (2014) provide preliminary evidence that the strength of association between maternal prenatal stress and infants' cognitive development varies based on the type of stress examined. Specifically, they found a weak but significant inverse association between maternal prenatal stress or anxiety and early child cognitive development (*r* = −.05), but the relationship between life‐events and cognitive development (*r* = −.31) was significantly greater than that for pregnancy‐related anxiety (*r* = −.08) or other subjective assessments of stress or anxiety unrelated to pregnancy (*r* = −.02). However, the outcome of interest in this meta‐analysis was restricted to the general intellectual skills of children aged 0 to 5, and did not examine the separate effects of perceived stress, stress response, trait anxiety, or state anxiety. Moreover, the number of studies examining the association between prenatal stress and children's cognitive development is growing rapidly and numerous relevant studies have been published since this meta‐analysis.

The strength of the association between maternal prenatal stress and children's later cognitive function also varies across studies, which may be explained by different approaches used to assess cognitive outcomes (i.e., direct observation; performance‐based assessment; and parent‐rated measures) in previous reviews. A growing body of work reveals a lack of correlation between these different measurement approaches, suggesting that pooling these cognitive measures may create a bias in the results (Acar et al., 2019; Miranda et al., 2015; O'Meagher et al., 2019).

The present review aimed to examine the relationship between prenatal maternal stress and anxiety and a range of key cognitive domains (i.e., general intellectual skills, attention, language, learning, memory, executive functions) in infancy, childhood, and adolescence (birth until 18 years of age). We also sought to examine the individual influence of the different aspects of stress and anxiety and type of cognitive measure used. We hypothesized that there would be a small negative association between maternal prenatal stress and offspring's cognitive domains. We also expected the strength of these associations to differ between the different psychological constructs (i.e., exposure to stressors, perceived stress, stress response, trait anxiety, state anxiety, and pregnancy‐related anxiety) and type of cognitive measurements (i.e., standardized performance‐based measures and questionnaires/observations), but to remain negative.

## METHOD

### Protocol and search strategy

The study protocol was registered on the National Institute of Health and Research PROSPERO International Prospective Register of Systematic Reviews (registration number: CRD42020185906). A search strategy was conducted for papers published before the date of the search (June 2021) on PsycINFO, MEDLINE, Web of Science Core Collection, CINAHL, and EMBASE databases. The search strategy included key‐terms related to the prenatal period, maternal anxiety, maternal stress, childhood, and cognitive development (see Table e1). The literature search was limited to humans and studies published in English. Additional articles were identified using reference lists from previous meta‐analyses and review articles.

### Inclusion and exclusion criteria

Prospective and retrospective observational studies investigating the association between symptoms of prenatal maternal anxiety and/or stress and children's cognitive outcomes were considered for inclusion. Measures of prenatal maternal anxiety and/or stress included validated self‐report questionnaires and physiological measures. The outcome measures were validated age‐standardized measures of general intelligence, learning, memory (long‐term free recall, long‐term recognition), language (receptive, expressive), speed of information processing, attention (span, divided, switching, sustained, selective), executive functions (working memory, inhibition, planning, organization, self‐monitoring, decision making, cognitive flexibility, word generation), and academic skills (reading, maths, spelling). Both direct assessment and parent and teacher‐rated questionnaires were included if relevant. There were no criteria for years of publication.

Studies were excluded for the following reasons: (i) the study was a treatment or clinical trial, case–control study, review, comment, letter, thesis, or book, (ii) individual cognitive skills were not assessed separately, (iii) the offspring were older than 18 years old, (iv) offspring were specifically selected based on a previous diagnosis (e.g., autism and attention‐deficit/hyperactivity disorder), (v) anxiety and/or stress was examined in the postnatal period, (vi) measures of anxiety and stress were combined in the analyses, (vii) children's cognitive function was assessed using experimental measures only, (viii) the study involved non‐human participants, and (ix) the study was not peer reviewed.

### Study selection

All the references retrieved using the systematic search strategy outlined above were downloaded and deduplicated into Endnote before being transferred, stored, and managed into Covidence. Titles and abstracts were screened independently by two reviewers, GD and SV. Full texts of potentially relevant articles were obtained and independently assessed for inclusion by the two reviewers using the previously described criteria. In case of disagreement, the reviewers discussed eligibility. When disagreement could not be resolved, PJA made the final decision. Ninety‐nine percent agreement was reached after the first screen of titles/abstracts, and 91% after the first screen of full texts. After discussion, 100% agreement was reached by the three reviewers.

### Data extraction

A customized extraction excel sheet adapted from the Cochrane Review handbook was used to guide extraction. Reviewers (GD and SV) extracted relevant information independently and discussion was organized in case of discrepancy. When available, extracted information included:

#### Independent variable

Measures used to assess maternal anxiety and/or stress during pregnancy; trimester(s) of exposition; type of cognitive assessment.

#### Outcome variables

Tasks; performance on cognitive tasks; unadjusted and adjusted effect sizes.

#### Confounding factors

Covariates considered and adjusted for in analysis.

#### Methodology

Participants' demographic information; participant attrition rate; study design; times of measurement, sample size.

### Bias and quality assessment

The quality of the studies selected was assessed by GD using a modified version of the Scottish Intercollegiate Guideline Network appraisal tool (SIGN) criteria for cohort studies (https://www.sign.ac.uk/sign‐50). SIGN is a widely used tool which appraises studies by examining six domains: study design, withdrawals and drop‐outs, potential for selection bias, measurement of outcomes and exposure factors, confounders and report of statistical analyses, and blinding. Using a scoring algorithm adapted from the Quality Assessment Tool for Quantitative Studies (http://www.ephpp.ca/tools.html), each of these domains were rated as being of weak, moderate, or strong quality, and an overall study quality rating was made based on the quality of each domain. Studies encompassing four or more strong and no weak domains were rated as strong; those with less than four strong and no more than one weak domain were rated as moderate; and those with more than two weak domains were rated as weak.

### Meta‐analytic procedures

Meta‐analyses were performed using the metafor package version 3.0‐2 in R (Viechtbauer, 2010), to estimate the pooled association between prenatal maternal stress and/or anxiety and children's different cognitive domains (i.e., general intellectual function, language, attention, learning and memory, and working memory). Subgroup analyses were then performed by stratifying results by type of exposure (i.e., stress and anxiety) and, when enough data were available, by type of anxiety and stress exposure (i.e., exposure to stressors, perceived stress, stress response, trait anxiety, state anxiety, and pregnancy‐related anxiety). When sufficient data were available, meta‐regressions were performed based on the type of cognitive assessment (i.e., standardized performance‐based measures or questionnaires/observations) and child's age (0–2 years old, 3–5 years old and 6 years old and older). Although the original intent was to assess associations for different timing of exposure, this was not possible due to the small number of eligible studies which assessed stress and/or anxiety at different timepoints during pregnancy and children of different age groups. Consequently, analyses were performed combining all trimesters of exposure and children from all age groups to increase power. Due to the limited number of studies including non‐White participants (<23%), ethnicity could not be used as moderator.

Given the variability in characteristics and sample sizes across studies, the studies' effect sizes (unadjusted correlation coefficient *r*) were pooled using a random‐effects model (Borenstein et al., 2010). The variance (*τ* ^2^) was estimated using the Hartung–Knapp–Sidik–Jonkman method, which provides more robust estimates when the number of studies is small (IntHout et al., 2014). Separate meta‐analyses were performed when at least three studies provided an effect for an outcome (Valentine et al., 2010). In situations where a study reported results for (i) different measures of a same cognitive domain, (ii) multiple types of stress and/or anxiety exposure, (iii) the same associations at different postnatal periods, and (iv) the same type of exposure at multiple time points during pregnancy, effect sizes were aggregated using inverse‐variance weighting taking into account their correlation. This correlation was based on the literature and sensitivity analyses were performed by using a range of possible correlations (Borenstein et al., 2021; Cheung, 2019). For each analysis, a correlational effect size *r* of .10 was considered as small, .30 as medium, and .50 as large (Cohen, 1988). The statistical significance level used was *α* = .05.

*I* ^2^ statistic was computed to assess for heterogeneity of effect sizes (Borenstein et al., 2011). *I* ^2^ reflects the percentage of variability in the effect size that is attributed to heterogeneity rather than sampling error. A value of 25% or below can be interpreted as a small amount of heterogeneity, while values of 50% and 75% or above represent moderate and high heterogeneity, respectively (Higgins & Thompson, 2002). For each analysis, we intended to generate funnel plots and perform rank test and Egger's test to investigate publication bias (Borenstein et al., 2021). The analytic codes can be found in Table e2.

## RESULTS

### Study selection, characteristics, and quality

**FIGURE 1:**
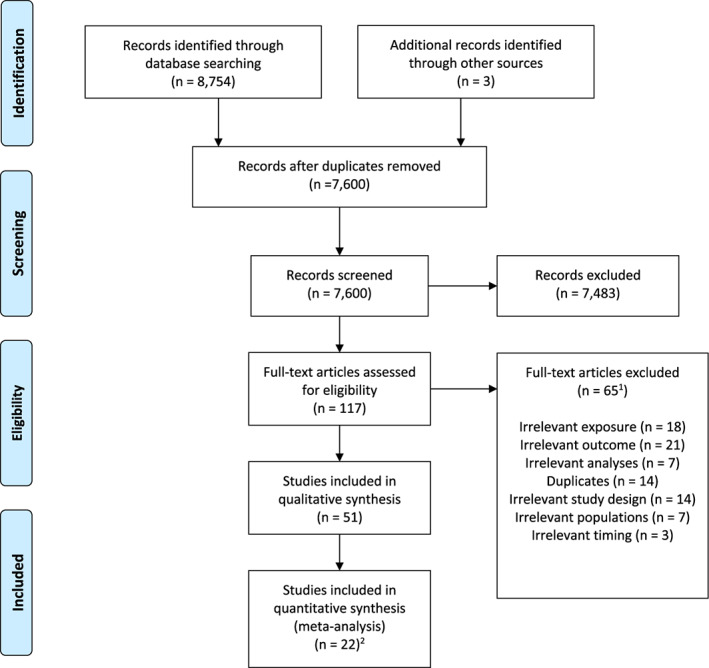
PRISMA study flow diagram for the systematic review. ^1^Some studies were excluded for more than one reason and thus reported more than once. ^2^Twenty‐nine studies were not included in the meta‐analysis because correlation coefficients could not be obtained from the published articles or authors.

The PRISMA flow diagram displayed in Figure 1 details the selection strategy and resulting outcomes. Our electronic search of five databases yielded 7600 articles after duplicates were removed and three articles were further identified from the references of the retrieved articles. Of these, 52 met our eligibility criteria and 22 had sufficient data that enabled their quantitative analysis. Characteristics of studies identified by the systematic search are reported in Tables 1 (stress) and 2 (anxiety). Thirty‐six studies examined the association between stress and children's cognitive outcomes and 25 investigated the influence of anxiety. Sample sizes ranged from 41 to 6979 in studies examining prenatal stress and from 43 to 6969 in those assessing prenatal anxiety. Of the eligible stress studies, the majority measured maternal prenatal perceived stress and exposure to stressors (*k* = 17 and *k* = 16), and a minority (*k* = 7) measured stress response. Of the eligible anxiety studies, *k* = 11 measured trait and state anxiety combined, *k* = 6 measured state anxiety, *k* = 6 measured trait anxiety, and *k* = 6 measured pregnancy‐related anxiety. Anxiety and stress were predominantly assessed once during pregnancy only, while 15 studies measured maternal prenatal stress and/or anxiety across multiple trimesters. Children were aged 0 to 11 years old. In relation to child cognition, most studies assessed offspring cognition during infancy, that is, 0–2 years old (56% and 52% of studies investigating stress and anxiety, respectively). There were 22 studies that met the inclusion criteria but sufficient data were not available from the published articles or authors. Table 3 lists eligible studies included in the analyses with their key demographic features and moderators, which varied from zero to 24 across studies. The mean number of participants was 23,307, with *M*_males_ 54%; *M*_ethnicity_ White 77%, Pacific 15%, African American/Black 10%, Middle Eastern 7%, and Eastern 8%. The key demographic features of the studies synthetized can be found in Table e3.

**TABLE 1:** Summary of eligible studies examining prenatal stress

Authors	*N*	Trimester of exposure	Timing of assessment	Aspect of stress	Cognitive domain	Children's age
Polanska et al. (2017)	219–337	T1	T1, T2, T3	Perceived stress	GIF, language	1–2 years
Nazzari et al. (2020)	104	T3	T3	Stress response	GIF	3 months
Lin et al. (2017)	225	T2‐T3	T3	Perceived stress	Attention, language	24–30 months
Keim et al. (2011)	358	T2	T2	Perceived stress	GIF, language	1 year
Huizink et al. (2002)	170	T1, T2, T3	T1, T2, T3	Perceived stress	Attention regulation	3–8 months
Gutteling et al. (2006)	113	T1, T2, T3	T1, T2, T3	Stress response, perceived stress	Learning and memory	6–7 years
DiPietro et al. (2006)	82–90	T3	T3	Perceived stress	GIF, attention	2 years
Davis et al. (2017)	91	T2, T3	T2, T3	Stress response	Visual skills, expressive language	6–9 years
D'Souza et al. (2019)	5768	T3	Late pregnancy	Perceived stress	Expressive language	2 years
Caparros‐Gonzalez et al. (2019)	41	T1, T2, T3	T1, T2, T3	Stress response	GIF, language	6 months
Campbell et al. (2019)	91	T3	T3	Stress response	Attention/concentration, memory	6.5 years
Bergman et al. (2007)	123	Pregnancy	14–19 months post‐pregnancy	Perceived stress	GIF	14–19 months
Austin et al. (2017)	125	Pregnancy	Pregnancy	Stressors	GIF, expressive language	2.5 years
Davis & Sandman (2010)	125	T1, T2, T3	T1, T2, T3	Perceived stress, stress response	GIF	3, 6 and 12 months
Gutteling et al. (2005)	119	Pregnancy	T1, T2, T3	Stressors, perceived stress	Attention	2 years
Huizink et al. (2003)	170	T1, T2, T3	T1, T2, T3	Perceived stress, stressors	Attention	3 and 8 months
Jensen et al. (2014)	6979	Pregnancy	Pregnancy	Stressors	GIF, attention, inhibition	8 years
Laplante et al. (2004)	58	T1, T2, T3	T1, T2, T3	Stressors	GIF	2 years
Laplante et al. (2008)	89	Pregnancy	Pregnancy	Stressors	GIF, language	5.5 years
Laplante et al. (2018)	103–122	Pregnancy	Pregnancy	Stressors	GIF, language	2.5 years
Moss et al. (2017)	145	Pregnancy	Pregnancy	Stressors	GIF	16 months
Simcock et al. (2017)	115	Pregnancy	Pregnancy	Stressors	Attention	2.5–4 years
Simcock et al. (2019)	118–134	Pregnancy	Pregnancy	Stressors	GIF	16 months
Neumann et al. (2019)	5202	Pregnancy	Pregnancy	Perceived stress	Language	4.5 years
LeWinn et al. (2009)	832 Collaborative Perinatal Project cohort	Pregnancy	T3	Stress response	GIF	7 years
Zhu et al. (2014)	152	T1, T2, T3	T1, T2, T3	Stressors	GIF, attention	16–18 months
Cortes Hidalgo et al. (2020)	4251	Pregnancy	20–25 weeks	Stressors	GIF	6 years
Chuang et al. (2011)	186	Pregnancy	Around birth	Stressors	Attention	2 years
Bhang et al. (2016)	641 MOCEH cohort	Pregnancy	T1, around delivery	Perceived stress	GIF, expressive language, problem solving	6 months
Slykerman et al. (2005)	550 ABC cohort	Pregnancy	T1, T3	Perceived stress	GIF	3.5 years
McDonald et al. (2016)	3360 AOB cohort	Pregnancy	Pregnancy	Perceived stress	GIF	1 year
Lamb et al. (2014)	3360	Pregnancy	Pregnancy	Perceived stress	GIF	7 and 11 years
Kim et al. (2015)	343	Pregnancy	Early or mid‐pregnancy	Stressors	GIF	6 and 12 months
Henrichs et al. (2011)	3139	Pregnancy	T2	Stressors	Language, non‐verbal GIF	1.5 and 2 years
Wu et al. (2021)	96	Pregnancy	T2, T3	Perceived stress	GIF	1.5 years
Plamondon et al. (2015)	112–150	T3	T3	Stressors	Focused and shifting attention, orientation, EF (spatial WM)	3 months–4 years

*Note*: T1 = trimester 1; T2 = Trimester 2; T3 = Trimester 3; General = general anxiety.

Abbreviations: EF, executive function; GIF, General Intellectual Functions; WM, working memory.

**TABLE 2:** Summary of eligible studies examining prenatal anxiety

Authors	*N*	Trimester of exposure	Timing of assessment	Type of anxiety (time of assessment)	Cognitive domain	Children's age
Savory et al. (2020)	58–76	T3	T3	Trait	GIF, Sustained attention, expressive and receptive language	1 year
Plamondon et al. (2015)	112–150	T3	T3	General	Focused and shifting attention, orientation, EF (spatial WM)	3 months–4 years
Pearson et al. (2016)	3270	T3	T3	General	Selective and switching attention, Processing speed, EF (WM)	8 years
O'Donnell et al. (2017)	6969	T3	T3	General	EF (WM)	8 years
Nazzari et al. (2020)	104	T3	T3	State	GIF	3 months
Koutra et al. (2017)	288	T3	T3	Trait	GIF, verbal, quantitative and perceptual skills, attention, memory	4 years
Koutra et al. (2013)	223	T3	T3	Trait	GIF, receptive and expressive language	18 months
Keim et al. (2011)	358	T1‐T2	T1‐T2	Trait	Visual skills, receptive and expressive language	1 year
Huizink et al. (2002)	170	T1, T2, T3	T1, T2, T3	PS	Attention regulation	8 months
Gutteling et al. (2006)	113	T1, T2, T3	T1, T2, T3	PS	Learning and memory	6–7 years
DiPietro et al. (2006)	82–90	T3	T3	State	GIF, attention regulation	2 years
Coplan et al. (2005)	46	T3	T3	State, trait	Attention span	3 months
Barker et al. (2011)	3298	T3	T3	General	Verbal skills	8 years
Davis & Sandman (2010)	125	T1, T2, T3	T1, T2, T3	PS, State	GIF	3, 6 and 12 months
Gutteling et al. (2005)	119	T1, T2, T3	T1, T2, T3	PS	Attention	2 years
Huizink et al. (2003)	170	T1, T2, T3	T1, T2, T3	PS	Attention	3 and 8 months
Buss et al. (2011)	86–87	T1, T2, T3	T1, T2, T3	PS, General	WM, inhibition	6–9 years
Clavarino et al. (2010)	428	Pregnancy	Pregnancy	General	Attention	5 and 14 years
Brouwers et al. (2001)	105	Pregnancy	T3	General	GIF	1 and 2 years
Vujović et al. (2019)	43	Pregnancy	T3	State, Trait	Language	3 years
Bolea‐Alamañac et al. (2018)	3845–4202	Pregnancy	T2‐T3	General	Attention	8.5 years
Ibanez et al. (2015)	1179–1268	Pregnancy	T2‐T3	State	GIF, language	2 and 3 years
Bendiksen et al. (2020)	1195	Pregnancy	T2, T3	General	Inattention	3.5 years
Irwin et al. (2020)	233	Pregnancy	T1, T2, T3	General	GIF, language	1 year
Wu et al. (2021)	96	Pregnancy	T2, T3	General	GIF	1.5 years

*Note*: T1 = trimester 1; T2 = Trimester 2; T3 = Trimester 3; General = general anxiety.

Abbreviations: EF, executive function; GIF, General Intellectual Functions; PS, pregnancy‐specific; WM, working memory.

**TABLE 3:** Tables of studies included in the meta‐analysis and their key demographic features

Authors	Stress evaluation	Time of stress assessment	*N*	Age	Cognitive outcomes	Outcome measure	Country	Sex (male, n)	Ethnicity	Effect size^a^	Reliability of outcome measure	Moderators
Simcock et al. (2017)	Stressors—Queensland Flood Objective Stress Score^b^	Pregnancy	115	6 months	Executive functions, language	Ages and Stages Questionnaire‐III	Australia	54	N/a	−0.11	Overall agreement for classifications = 92%; correlation coefficients: .75–.82	Depression during pregnancy, current depression, anxiety and stress, maternal marital status, SES, infant birth size (gestational age and birth weight)
Simcock et al. (2019)	Stressors—Queensland Flood Objective Stress Score^b^	Pregnancy	118–134	2.5 and 4 years	Attention	Child Behavior Checklist	Australia	66.5^c^	97.6% White^c^ 2.5% n/a	0.05	Criterion‐related validity *r* = .78	Gestational age at birth, birthweight, child sex, child age
Savory et al. (2020)	Stait‐Trait Anxiety Inventory	Birth	76	1 year	IQ, language, attention	Bayley scales of infant and toddler development; Carousel Toy	UK	46^g^	96% White 4% n/a	−0.19	Bayley: Average reliability coefficient: .91 Carousel: n/a	Infant age at questionnaire completion, parity
Plamondon (2015)	Stressors—Number of stressful events; Stait‐Trait Anxiety Inventory	T3	112–165	18 months and 4 years	Attention, working memory	Infant behavior Questionnaire and CANTAB	Canada	137^g^	90% White 3% mixed ethnicity 2% African 1.5% Hispanic 1% East Indian	0.01	Chronbach's *α*: .71–.88	Child sex, birthweight, and gestational age
Pearson et al. (2016)	Anxiety—Crown‐Crisp index	18–32 (*M* = 25)^d^	3624	8 years	Working memory	Wechsler Intelligence Scale for Children and Non‐words	UK	1822^g^	96% White^g^ 4% n/a	−0.04	Wechsler: test–retest reliability: .80–.89 Non‐words: n/a	Postnatal depression
Nazzari et al. (2020)	Anxiety—State–Trait Anxiety Inventory; stress‐ diurnal cortisol levels	T3	104	3 months	General intellectual function	Bayley scales of infant and toddler development	Italy	53	97.2% (Italian) 2.8% n/a	−0.18	Average reliability coefficient: .91	Maternal age, education, socioeconomic status, parity, infants' age, mode of delivery, length of labor, gestational age, birth weight, head circumference, actual weight, postnatal smoke exposure, breastfeeding versus Formula‐feeding, maternal emotional availability and IQ, gestational age at birth
Moss et al. (2017)	Stress—the Queensland Flood Objective Stress Scale^b^	Pregnancy	145	1 year	General intellectual function	Bayley scales of infant and toddler development	Australia	81	96% White 4% n/a	−0.01	Average reliability coefficient: .91	Other major life events than the flood, parenting stress, postnatal anxiety, stress and depression, birth outcome data (birth weight and gestation)
Koutra et al. (2017)	Anxiety—State–Trait Anxiety Inventory (Trait scale)	T3	288	4 years	General intellectual function; attention	Mccarthy Scales of Children's Abilities; Attention Deficit Hyperactivity Disorder Test (Questionnaire)	Greece	160	95.5% (Greek) 4.5% n/a	0.00	Mccarthy Scales of Children's Abilities: n/a Questionnaire: Cronbach's *α*: .83	Maternal age at delivery, maternal education, smoking status at 4 years, working status at 4 years assessment, child's sex, prematurity, breastfeeding duration, pre‐school attendance, TV watching, birth order at 4 years, number of children in the family, quality of assessment and examiner
Koutra et al. (2013)	Anxiety—State–Trait Anxiety Inventory (Trait scale)	T3	223	1.5 year	Language	Bayley scales of infant and toddler development	Greece	125	97.3% (Greek) 2.7% n/a	0.04	Average reliability coefficient: .91	Maternal age at delivery, maternal education, smoking status at 4 years, working status at 4 years assessment, child's sex, prematurity, breastfeeding duration, pre‐school attendance, TV watching, birth order at 4 years, number of children in the family, quality of assessment and examiner
Keim et al. (2011)	Anxiety—State–Trait Anxiety Inventory	T1‐T2	358	1 year	Non‐verbal intellectual skills, language	Mullen Scales of Early Learning	USA	193	N/a	−0.02	Internal consistency reliability: 0.75–0.83	Income, pre‐pregnancy body mass index (BMI), education, social support, self‐esteem, maternal age, infant sex, gestational age, presence of a spouse/partner, depressive symptoms, and perceived stress
Laplante et al. (2018)	Stress—Iowa Flood 100^b^	T1, T2, T3	132	2.5 years	General intellectual function; language	Bayley scales of infant and toddler development; mccarthy Scales of Children's Abilities	USA	69	100% White	−0.11	Bayley: Average reliability coefficient: .91 Mccarthy: n/a	Positive mental health status of women during pregnancy, SES, maternal exposure to other life events than the flood, maternal social support at 30 months, parenting stress, current maternal stress, infants' gestational age at birth and birth weight, medical and obstetric history (e.g., cigarette/alcohol use, hypertension, diabetes, infections)
Laplante et al. (2008)	Stress—Storm32 score^b^; Life Experiences Survey	T1, T2, T3	89	5.5 years	General intellectual function; language	Wechsler Preschool and Primary Scale of Intelligence‐Revised and Peabody Picture Vocabulary Test—Revised	Canada	42	100% White	−0.34	Criterion‐related validity *r*: .92	Maternal age and education, marital status, parental job classification, household income, number of obstetric complications, including alcohol and cigarette use, children's birth weight, birth length, and gestational age
Huizink et al. (2002)	Stress—Daily hassles; Anxiety—Pregnancy‐Related Anxiety Questionnaire‐Revised	T1, T2, T3^e^	170	3–8 months	Attention regulation	Infant behavior Questionnaire	Netherland	84	96% White 4% n/a	−0.11	The interrater reliability: >0.85	Educational level and professional level of the pregnant woman and her partner, pregnancy complications, use of medication during pregnancy, medication that poses risks for the fetus, fertility problems, high blood pressure, in vitro fertilization, gestational diabetes mellitus, gynecological risk, pre‐existing disease, smoking during pregnancy, alcohol use during pregnancy, birth weight, gestational age at birth, complications during delivery, gender, breast‐feeding, psychological well‐being, and perceived stress of the mother at 3 and 8 months after childbirth and postnatal depression scores of the mother
Cortes Hidalgo et al. (2020)	Stress—Cecil and colleagues' (2014) stress exposure construct	T2	4251	6 years	Non‐verbal intellectual skills	Snijders‐Oomen Nietverbale intelligentie test, 2.5–7‐ revise	Netherland	2057	69% White 10% Caribbean 12% Moroccan/Turkish 5% African 4% Indonesian	−0.14	Average alpha reliability: .90	Maternal IQ score, maternal and paternal educational level, family income, maternal alcohol consumption, and smoking during pregnancy
Jensen et al. (2014)	Stress—Measure of interprersonal stress^b^	T2	6979	8 years	Non‐verbal intellectual skills	WISC‐IV; teach	UK	N/a	N/a	−0.03	WISC: reliability coefficient: .97 Teach: n/a	N/a
Gutteling et al. (2006)	Stress—Everyday Problem List and cortisol saliva; Anxiety—Pregnancy‐Related Anxiety Questionnaire‐Revised	T1, T2, T3	112	6 years	Learning and Memory	Test of Memory and Learning	Netherland	50	N/a	0.00	Cronbach's *α*: above .74	Child's gender, gestational age, and birthweight, maternal age, maternal educational level, prenatal smoking (yes/no) and alcohol use (yes/no), and postnatal maternal stress
DiPietro et al. (2006)	Anxiety—Pregnancy Experience Scale; Anxiety subscale of the Profile of Moods Scale; Spielberger State–Trait Anxiety Scales. Stress—Daily Stress Inventory; Perceived Stress Scale	T3	94	2 years	General intellectual function; attention	Infant Behavior Record	USA	46^g^	85% White 12% African American 3% Asian	0.15	Interobserver reliability *k*s: .82 to .85	Maternal education, fetal sex, prenatal depression and stress, postnatal depression, and stress
Coplan et al. (2005)	Anxiety—Stait‐Trait Anxiety Inventory	T3	60	3 months	Attention span	Infant behavior Questionnaire	Canada	N/a	97% White^g^ 3% n/a	−0.25	N/a	Trait anxiety, maternal age, parental education, pregnancy complications, smoking and drinking habits during pregnancy, birth order of the unborn child, infant birth weight, length of infant
D'Souza et al. (2019)	Stress—Perceived Stress Scale	T3	5768	2 years	Language	Mccarthy Scales of Children's Abilities	New‐Zealand	2964^g^	67% White^f^ ^,^ ^g^ 24% Māori 16% Asian 21% Pacific 3% Middle Eastern, Latin American or African 7% Other	−0.10	Reliability coefficient: .90	Mother's ethnicity and education, mother's age when pregnant, area‐level deprivation, child's gender, gestational age, birthweight, and age at assessment
Campbell et al. (2019)	Stress—Salivary cortisol	T3	242–244	6.5 years	Memory, attention	Wide Range Assessment of Memory and Learning	USA	133	60% Hispanic^g^ 28% Black 9% White 4% Other or multiple ethnic groups	0.05	N/a	Maternal age, race/ethnicity, education completed, self‐reported pre‐pregnancy weight and height; child sex, date of birth, and gestational age at birth; gestational age at cortisol assessment
Bergman et al. (2007)	Stress—26‐item Stressful Life Events Questionnaire, adapted from the Inventory of Ranked Life Events	Postnatal visit	123	1–1.5 years	General intellectual function	Bayley scales of infant and toddler development	UK	60	84% White 6% Asian‐Indian/subcontinent 8% Black 2% Middle Eastern	−0.41	Interrater reliability: 0.91	Maternal age, parity, ethnicity, smoking, alcohol, and prescribed drugs during pregnancy; birth weight, gestational age at birth, method of delivery, and child sex; maternal postnatal anxiety and depression and maternal social support postnatally
Buss et al. (2011)	Anxiety—pregnancy‐related anxiety scale; state–trait anxiety inventory (state scale)	T1, T2, T3	89	6–9 years	Executive functions (Working memory and inhibitory control)	Flanker task; Corsi block‐tapping test	USA	50	77% White 7% African American 14% Asian 2% Other	−0.05	STAI: Cronbach's *α*: .92 Pregnancy‐related anxiety scale: Cronbach's *α*: .75–.85	Maternal (race/ethnicity, obstetric risk, parity, age at delivery, postpartum and concurrent depression, and WAIS PRI score) and child (GA at birth, sex, age at assessment) variables

*Note*: T1 = Trimester 1; T2 = Trimester 2; T3 = Trimester 3.

^a^ Based on IQ when available.

^b^ Scales designed for the study.

^c^ Average of 2 and 4 years old.

^d^ In week.

^e^ Only prenatal stress at T1 was included in the analyses.

^f^ Mother could identify themselves with multiple ethnicities.

^g^ Based on the entire sample.

In relation to study quality, two studies were classified as strong, 18 as moderate, and 2 as weak (Table 4). Few studies reported information on whether the outcome assessment was made blind to the exposure status (*k* = 14), which was needed to reach an overall strong quality rating. A notable risk of bias was also identified, related to inappropriate adjustment of missing data when attrition rate was high. Notably, all studies addressed a specific research question, clearly defined their outcome of interest, and used reliable and valid methods of measurement. Publication bias could not be assessed as there were too few studies to properly assess a funnel plot, rank test, or Egger's test (Rothstein et al., 2006).

**TABLE 4:** Summary of quality of studies included in meta‐analysis

Study	Design	Selection bias	Withdrawals	Confounder and analyses	Measurement	Blinding	Overall study quality
Savory et al. (2020)	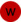	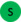	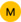	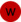	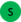	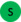	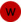
Plamondon et al. (2015)	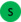	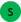	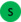	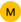	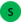	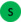	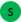
Pearson et al. (2016)	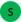	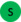	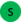	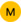	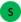	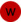	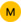
Nazzari et al. (2020)	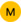	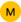	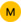	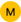	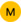	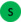	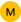
Koutra et al. (2017)	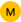	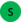	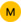	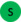	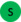	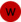	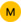
Huizink et al. (2002)	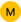	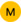	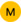	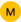	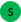	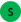	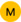
Gutteling et al. (2006)	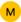	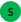	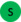	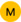	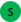	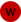	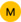
DiPietro et al. (2006)	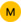	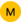	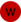	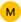	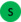	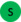	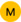
Coplan et al. (2005)	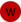	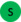	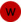	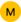	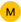	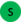	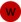
D'Souza et al. (2019)	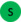	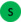	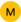	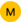	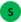	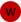	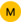
Campbell et al. (2019)	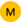	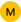	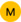	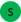	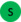	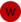	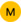
Bergman et al. (2007)	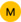	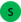	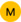	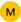	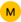	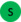	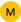
Simcock et al. (2017)	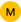	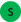	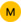	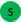	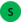	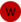	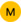
Simcock et al. (2019)	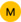	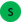	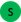	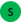	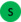	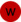	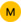
Laplante et al. (2018)	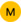	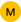	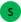	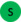	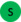	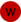	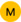
Laplante et al. (2008)	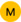	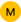	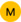	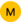	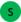	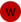	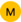
Jensen et al. (2014)	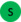	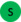	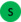	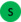	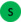	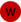	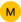
Moss et al. (2017)	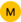	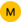	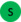	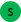	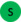	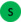	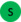
Buss et al. (2011)	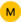	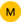	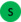	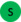	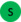	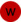	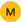
Koutra et al. (2013)	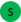	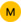	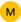	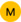	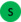	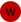	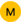
Keim et al. (2011)	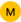	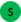	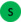	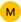	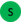	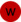	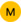
Cortes Hidalgo et al. (2020)	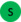	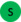	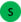	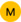	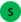	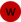	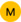

### Maternal prenatal anxiety and stress combined (Table 5, Figure 2)

**TABLE 5:** Association between prenatal stress and anxiety, stress, anxiety, and offspring cognitive outcomes across all ages of development

Outcome	Stress and anxiety			Anxiety			Stress		
	Number of studies (sample size)	Pooled correlations	*I* ^2^ value, %	Number of studies (sample size)	Pooled correlations	*I* ^2^ value, %	Number of studies (sample size)	Pooled correlations	*I* ^2^ value, %
		*r* (95% CI), *p*‐value			*r* (95% CI), *p*‐value			*r* (95% CI), *p*‐value	
General intellectual skills	10 (*n* = 8683)	−.12 (−.24; −.003), .045	94.02	4 (*n* = 550)	−.005 (−.28; .27), .96	74.24	8 (*n* = 8319)	−.15 (−.29; −.01), .04	94.29
Attention	8 (*n* = 7605)	−.04 (−.11; .03), .39	67.98	5 (*n* = 671)	−.08 (−.21; .06), .19	49.80	5 (*n* = 7202)	.01 (−.01; .02), .33	2.69
Expressive language	6 (*n* = 6644)	−.03 (−.15; .09), .54	77.11	—	—	—	—	—	—
Receptive language	5 (*n* = 852)	−.12 (−.34; .09), .18	78.54	—	—	—	—	—	—
Overall language	7 (*n* = 6734)	−.09 (−.22; .04), .15	86.57	3 (*n* = 640)	−.06 (−.45; .33), .57	82.59	4 (*n* = 6094)	−.12 (−.37; .13), .24	81.83
Learning and memory	3 (*n* = 546)	−.007 (−.30; .29), .93	73.35	—	—	—	3 (*n* = 546)	.01 (−.26; .28), .84	62.98
Executive functions	5 (*n* = 10,670)	−.03 (−.11; .05), .35	83.58	3 (*n* = 3823)	−.04 (−.06; −.01), .02	1.29	3 (*n* = 6960)	−.01 (−.39; .36), .88	79.48

*Note*: —,not applicable (not enough studies to run meta‐analysis).

**FIGURE 2:**
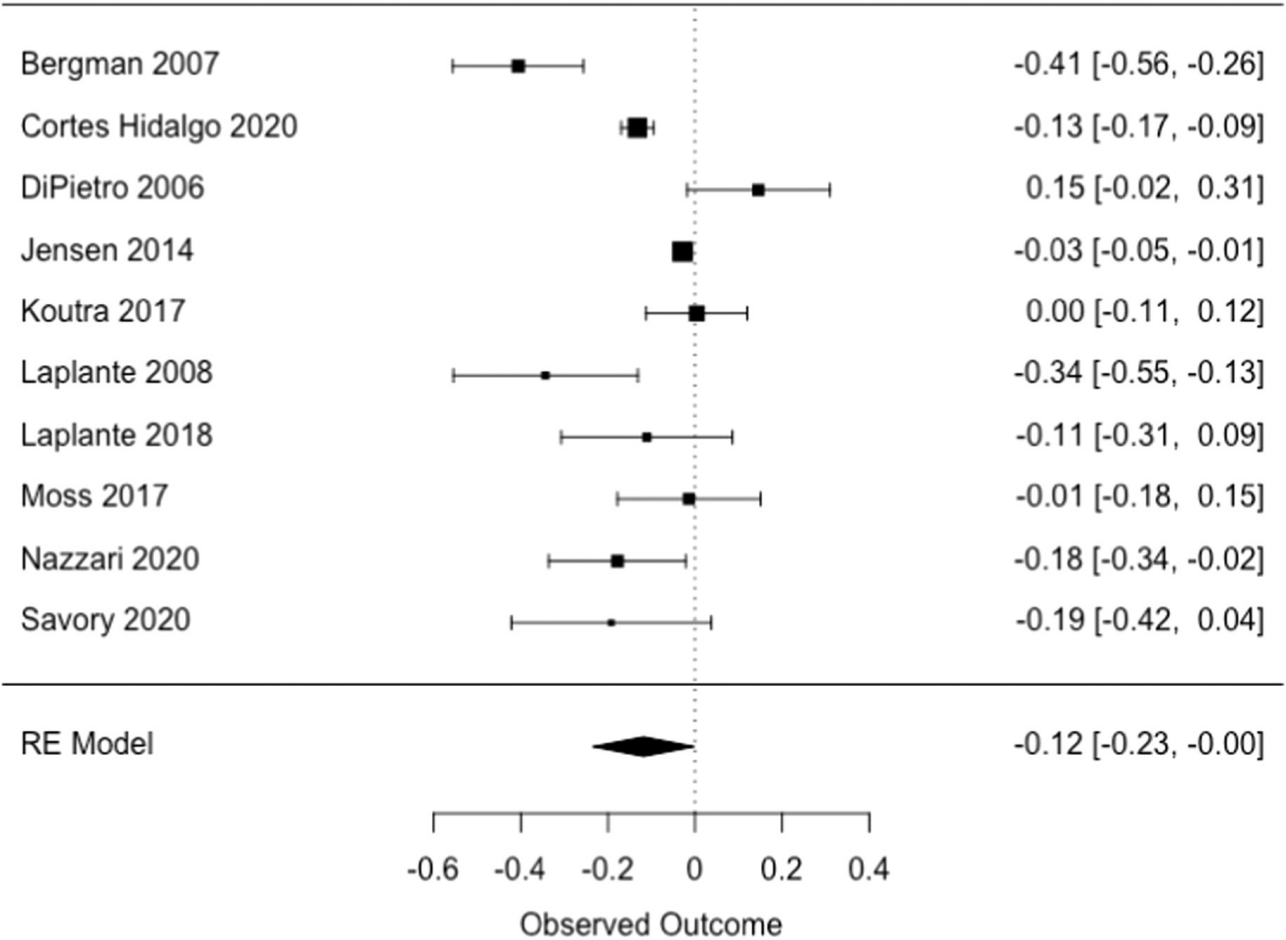
Association between stress and/or anxiety and general intellectual skills in offspring.

Exposure to prenatal maternal stress and/or anxiety was significantly associated with lower general intellectual skills (*r* = −.12 [95% CI, −.24; −.005], *I* ^2^ = 94%), albeit with a small effect size (Figure 2). Nonsignificant negative associations were observed between prenatal exposure to maternal stress and/or anxiety and children's executive function, attention, learning and memory, and language skills (Table 5). Similar results were obtained when different coefficients of correlation were used for the aggregation of multiple effect sizes within some studies (Borenstein et al., 2010)—that is, sensitivity analyses. Heterogeneity across studies varied from moderate (67%) to high (94%).

### Maternal prenatal anxiety (Tables 5 and 6; Figure 3)

**TABLE 6:** Summary of studies examining different types of prenatal maternal exposures

Studies	Stress	Anxiety
Polanska et al. (2017)	Perceived stress *but not* exposure to stressors was negatively associated with child intellectual development at age 2	—
Laplante et al. (2004)	Exposure to stressors *but not* perceived stress was negatively associated with general intellectual skills, receptive language, and expressive language at age 2	—
Bergman et al. (2007)	Both perceived stress *and* exposure to stressors were associated with lower general intellectual skills in infants	—
Davis & Sandman (2010)	Maternal stress response *but not* perceived stress was associated with the trajectory of infant development and influenced cognitive functioning at 1 year of age	Pregnancy‐specific anxiety *but not* state anxiety was associated with the trajectory of infant development and influenced cognitive functioning at 1 year of age
Huizink et al. (2002)	Perceived stress *but not* exposure to stressors was associated with lower attention skills in the offspring	Pregnancy‐related anxiety *was* associated with lower attention skills in offspring
Huizink et al. (2003)	Perceived stress *but not* exposure to stressors was associated with lower attention in children	Pregnancy‐related anxiety *was* associated with lower attention in children
DiPietro et al. (2006)	Exposure to stressors *but not* perceived stress was associated with lower general intellectual skills in offspring	General anxiety *but not* state anxiety was associated with lower general intellectual skills in offspring
Coplan et al. (2005)	—	State anxiety *but not* trait anxiety was associated with lower attention span in infants
Buss et al. (2011)	—	Pregnancy‐related anxiety *but not* state anxiety was associated with lower performances on the flanker task. Both types of anxiety were associated with lower performances on a sequential memory task
Gutteling et al. (2006)	Exposure to stressors *but not* perceived stress was negatively associated with attention at age 6	Pregnancy‐related anxiety *was not* associated with attention at age 6
Gutteling et al. (2005)	Exposure to stressors *was not* associated with children's attention skills	Pregnancy‐related anxiety *was* associated with lower attention skills in offspring
Keim et al. (2011)	Perceived stress *was* associated with lower intellectual skills in offspring	Trait anxiety *was* not associated with intellectual skills in offspring
Nazzari et al. (2020)	Stress response *was* negatively associated with infant cognitive composite scores	Trait anxiety *was not* associated with infant cognitive composite scores
Plamondon (2015)	Exposure to stressors *was* not associated with attention or working memory in offspring	General anxiety *was not* associated with attention or working memory in offspring
Wu et al. (2021)	Stress *was* negatively associated with general cognitive skills in infants	Anxiety *was* negatively associated with general cognitive skills in infants

**FIGURE 3:**
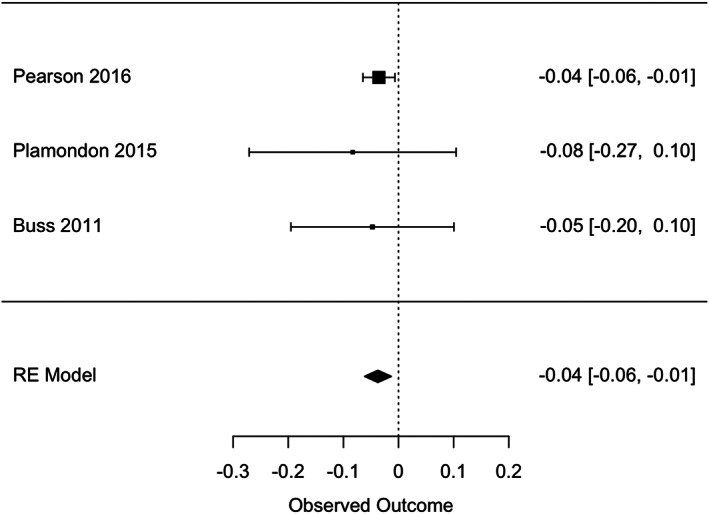
Association between prenatal exposure to anxiety and executive functions in offspring.

Exploratory analyses of three studies revealed a weak negative association between prenatal exposure to anxiety and later executive functions in the offspring (*r* = −.04, 95% CI [−.06; −.01], *I* ^2^ = 1%; Figure 3). No significant associations were found between maternal prenatal anxiety and offspring's general intellectual skills, attention, and language abilities (Table 5). Similar results were obtained in our sensitivity analyses. Notably, these analyses included a small number of studies (3–5) of which some had small sample sizes, warranting cautious interpretation of these results. Heterogeneity was high, except for executive functions. Further quantitative analyses according to the type of anxiety were limited by the small number of studies. Qualitative analysis revealed a pattern of distinct results based on the type of prenatal exposure (Table 6; Buss et al., 2011; Coplan et al., 2005; Davis & Sandman, 2010; DiPietro et al., 2006). Specifically, the authors compared the effects of different types of anxiety and all found differential effects. For instance, Coplan et al. (2005) indicated that state but not trait anxiety was associated with children's attention span, and Davis & Sandman (2010) reported that pregnancy‐related anxiety but not state anxiety was associated with infants' neurodevelopment.

#### Maternal prenatal stress (Tables 5 and 6; Table e4; Figure 4)

**FIGURE 4:**
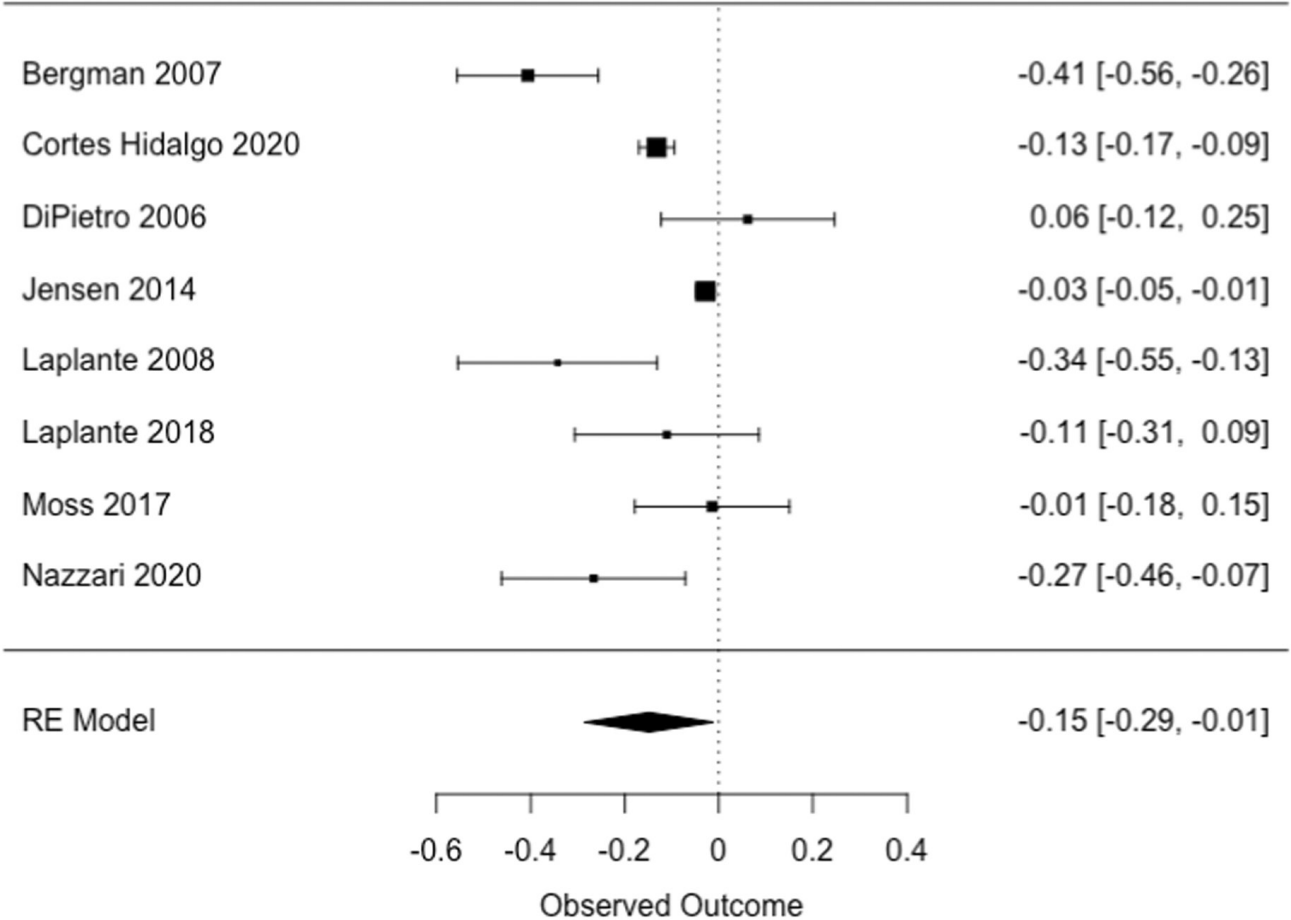
Association between prenatal exposure to stress and general intellectual skills in offspring.

A weak negative relation was found between maternal prenatal exposure to all types of stress combined (hereafter referred to as general stress) and offspring's general intellectual skills (*r* = −.15 [95% CI, −.29; −.01], *I* ^2^ = 94%; Figure 4). In contrast, no evidence for an association was found between prenatal exposure to maternal general stress and children's attention, learning and memory abilities, and executive functions (Table 5). When examining the relation between prenatal exposure to stressors and children's general intellectual skills, attention and language abilities, no significant association was found (Table e4). Similar findings were obtained in sensitivity analyses. Heterogeneity was high, except for attention. Sample sizes also varied, with three to eight studies (*n* = 546–8319) for the primary analyses (i.e., association between stress and/or anxiety and children's cognitive development) and three to seven studies (n = 326–7127) for the secondary analyses (i.e., association between the different types of stress and anxiety and children's cognitive development). A lack of studies on perceived stress and stress response prevented further quantitative analysis. Twelve of 15 studies that studied multiple types of exposures found that effects differed between types of exposures (Table 6). The direction of these associations, however, was inconsistent. For instance, some studies showed that prenatal exposure to stressors but not perceived stress negatively impacted children's cognitive development (Gutteling et al., 2006; Laplante et al., 2004), while other studies showed the opposite (DiPietro et al., 2006; Huizink et al., 2002, 2003; Polanska et al., 2017).

### Moderation analyses

#### Cognitive outcome measurement (standardized performance‐based instrument vs. questionnaire)

There was no evidence from the meta‐regression that meta‐analytic effects were moderated by children's type of attention measure (*r* = −.03, 95% CI [−.10; .03], *p* = .33, *I* ^2^ = 40.4%). Notably, only one moderation analysis could be conducted because there were often fewer than three effects per level of each moderator. Owing to low power, results should be interpreted cautiously.

#### Child age

There was no evidence from the meta‐regression that the relationship between children's general intellectual skills and attention was moderated by their developmental stage (Table 7). Such relationships with other cognitive domains could not be examined due to an insufficient number of studies. We note, results should be interpreted cautiously due to low power and high heterogeneity between studies.

**TABLE 7:** Association between Children's developmental stage and cognitive outcomes

Outcome	Developmental stage	Number of studies (sample size)	Correlations	*I* ^2^ value, %
			*r* (95% CI)	
General intellectual skills	Toddler (0–2 years old)	4 (530)	−.13 (−.39; .13)	82.76
	Child (3–5 years old)	2 (391)	−.03 (−.71; .64)	24.04
	Older child (6 years and older)	3 (7187)	−.14 (−.51; .22)	97.45
Attention	Toddler (0–2 years old)	4 (440)	−.81 (−.23; .07)	47.94
	Child (3–5 years old)	2 (414)	−.06 (−1.44; 1.32)	78.98
	Older child (6 years and older)	3 (6921)	−.14 (−.51; .22)	97.45

#### Child sex

There was no evidence from the meta‐regression that the relationship between children's general intellectual skills (*t*(8) = −.003, 95% CI [−.01; .008]) and attention (*t*(5) = −.006, 95%[−.02; .007]) were moderated by their sex.

#### Timing

A qualitative analysis of the potential moderating impact of timing on the relationship between prenatal maternal stress and children's later cognitive development suggested that earlier exposures resulted in more detrimental effects than later exposures (Buss et al., 2011; Davis & Sandman, 2010; Gutteling et al., 2006; King & Laplante, 2005; Laplante et al., 2018). Gutteling et al. (2006), for instance, showed that exposure to greater levels of stressors early but not late in pregnancy was associated with lower attentional skills in children. Similarly, Davis & Sandman (2010) reported that elevated levels of maternal pregnancy‐specific anxiety early but not late in gestation were associated with lower mental development in the offspring. Furthermore, King and Laplante (2005) found that exposure to stressors during the first and second but not the third trimester was associated with lower intellectual abilities in infants.

## DISCUSSION

The present meta‐analysis examined the association between maternal prenatal stress and anxiety and offspring's cognitive development, while considering the different definitions of stress and cognitive measures used in the literature. Although previous reviews have examined such relationship in infants, this meta‐analysis builds on previous findings and offers four unique contributions by focusing on (i) distinct types of stress and anxiety, (ii) specific cognitive domains, (iii) both infants and children aged up to 18 years old, and (iv) different types of cognitive measurements.

Two key findings emerged from this study. First, there was a weak negative association between the exposure to stress and anxiety (combined) during pregnancy and the offspring's general intellectual development. These findings are in line with those of previous systematic reviews and meta‐analyses which reported a weak negative association between maternal prenatal stress and/or anxiety and infants' general intellectual skills (Kingston et al., 2015; Rogers et al., 2020; Tarabulsy et al., 2014). Our findings further suggest that meaningful associations did not extend to other cognitive skills, such as language, attention, and executive functions. This contrasts with Rogers and colleagues' (2020) meta‐analysis which reported a weak negative association between maternal prenatal stress and/or anxiety and children's language function (*r* = −.11; Rogers et al., 2020). Different factors may contribute to this disparity, including distinct selection criteria around the type of prenatal exposure and outcome measures. The inconsistent control of confounding factors found in studies included in the meta‐analyses is also likely to contribute to the distinct findings. Moreover, results could have differed due to the inclusion of additional studies published after 2020 in this meta‐analysis.

The second key finding of this meta‐analysis was that most studies which examined different types of prenatal maternal stress and/or anxiety exposure reported differential effects on children's cognitive skills. While our meta‐analysis also showed a negative effect of anxiety but not general stress on executive functions and a negative effect of general stress but not anxiety on children's general intellectual skills, these differences could not be quantified due to a lack of studies. Based on this preliminary evidence, we argue that future studies should examine these prenatal types of exposure independently to better understand the association between maternal prenatal stress and/or anxiety and children's cognitive development.

Findings from performance‐based measures and questionnaires/observations were similar. Analyses, however, were limited and may have lacked power due to the small number of studies included. Accumulating evidence in the literature suggests an absence of strong correlations between parent ratings, observers, teacher‐ratings and performance‐based measures at all ages (Acar et al., 2019; Cho et al., 2011; McAuley et al., 2010). While reasons for the apparent dissociation between different cognitive measures remain poorly understood, it has been suggested that the different types of cognitive measures assess different aspects of the same underlying construct (Anderson et al., 2002). Discrepancies between these measures might also stem from differences in psychometric properties and ecological validity (Wallisch et al., 2018). The existing literature raises questions about the pooling of different types of cognitive assessments to investigate children's cognitive function. Future reviews should thus aim to separate different measures of cognition when examining the association between maternal prenatal stress and/or anxiety and children's later cognitive function.

Preliminary findings showed that the influence of maternal prenatal stress and/or anxiety on children's cognitive function did not vary based on children's developmental stage or sex. While these findings concur with those of Tarabulsy et al. (2014) and suggest that the influence of maternal prenatal stress and/or anxiety on children's cognitive function is long‐lasting, these findings must be interpreted with caution due to the low power and high heterogeneity found in the analysis (Guolo & Varin, 2017; Higgins & Thompson, 2004).

Decades of research have demonstrated that the timing of prenatal stress and/or anxiety was important to take into account when examining child outcomes (Epel et al., 2018; Roesch et al., 2004; Sandman et al., 2012). With regard to children's general cognitive abilities more specifically, Tarabulsy et al. (2014) reported that a negative association was only found when exposure occurred during the 3rd trimester. The timing of prenatal maternal stress and/or anxiety could not be assessed quantitatively in the present study due to the small number of eligible studies analyzing these psychological constructs at different timepoints, so we did a preliminary qualitative analysis of studies which systematically evaluated the impact of timing across several trimesters. Our findings suggest that children exposed to stress and/or anxiety early during pregnancy exhibit worse cognitive outcomes than those exposed to these emotional reactions states late in pregnancy (Buss et al., 2011; Davis & Sandman, 2010; Gutteling et al., 2006; King & Laplante, 2005; Laplante et al., 2018), which contrast with findings from Tarabulsy et al.' (2014). More research, however, is needed to ascertain this assumption, which is only based on five studies.

The present meta‐analysis has several limitations that need to be taken into account when interpreting the results. One important consideration is that our analyses were correlational. As such, causality between maternal prenatal stress and/or anxiety and adverse children's cognitive outcomes cannot be established, and the role of covariates in this relationship remains unknown. It is likely that factors that were not taken into account in the meta‐analysis (e.g., maternal cognitive abilities, children's postnatal mental health, parental emotional support, family socioeconomic status, maternal prenatal depression, and maternal postnatal mental health) played an important role in the relationship between prenatal maternal stress and/or anxiety and children's later cognitive functions (Anger & Heineck, 2010; Duncan & Magnuson, 2012; Hubbs‐Tait et al., 2002; Kingston et al., 2015; Rogers et al., 2020). Meta‐analyzing effects adjusted for confounders, however, were considered implausible given the extensive variability in confounders included across studies and lack of common set of covariates examined. Another limitation of this study was the collapsing of participants across all age groups in the main analyses. Cognitive skills continue to develop throughout childhood and adolescence (Cromer et al., 2015; Reber, 1986). Consequently, children prenatally exposed to stress and/or anxiety may exhibit different cognitive difficulties based on their developmental stage, a difference which is likely to be masked when children from all age groups are combined. A third limitation of this body of work is that some of the pooled estimates were based on a limited number of studies, which also sometimes had a small sample size. Although we used an approach that is more robust in cases of small numbers of studies and performed sensitivity analyses, this may have increased heterogeneity in our analyses and resulted in an overestimation of some of our effect sizes (Turner et al., 2013). Indeed, moderate to high heterogeneity was found across studies, potentially limiting our ability to detect meaningful relationships (Higgins & Green, 2011). Alternatively, this high heterogeneity might result from the wide range of measures used to assess stress and anxiety across studies. Indeed, these measures were found to have varying psychometric properties and be differently associated with one another and with children's cognitive outcomes, making these instruments not interchangeable (Cieszyński et al., 2020; Epel et al., 2018; Graignic‐Philippe et al., 2014; Nast et al., 2013; Short et al., 2016; Vanaelst et al., 2012). Lastly, due to the sampling of the studies included in this meta‐analysis, our findings are mainly based on studies conducted with White populations. While this may increase homogeneity within the included studies, the findings may not generalize to other racial groups (Ball & Crawford, 2005).

Thus, we argue that more studies are required to draw firm conclusions about the association between prenatal maternal stress and/or anxiety and children's cognitive development. Future studies should aim to separately examine the influence of the different aspects of stress and anxiety on children's later cognitive skills. Maternal stress and anxiety should be measured at multiple timepoints during pregnancy, and the association between each of these types of exposure and children's later cognitive functions at each timepoint should be examined independently. Future studies should also aim to increase consistency in the covariates included in the analyses to enable meta‐analyses to examine adjusted associations between maternal prenatal stress and/or anxiety and children's later cognitive development. For instance, key variables consistently found to influence the relationship between maternal prenatal stress and/or anxiety and children's later cognitive development should be identified and included in future studies, with any additional covariate considered being added in a step‐wise manner. This is needed to inform prevention and intervention strategies to support pregnant women experiencing or at risk of experiencing stress and/or anxiety. Notably, researchers should not only examine the general intellectual skills of infants but also extend their focus to more specific cognitive domains of both children and adolescents. Moreover, accumulative evidence supports the deleterious effects of co‐occurring risk factors on child developmental outcomes (Appleyard et al., 2005; Calderón‐Garcidueñas et al., 2016; Pawlby et al., 2011). Given that the different aspects of stress and anxiety often co‐occur, another reasonable next step, in this line of research, is to explore potential differences in the cognitive function of children prenatally and postnatally exposed to one or several of these constructs (Konstantopoulou et al., 2020; Schneiderman et al., 2005).

In summary, our findings support the current consensus that prenatal maternal stress and/or anxiety is likely to be negatively, albeit weakly, associated with offspring cognitive function. These findings are of public health significance and support the need for screening and interventions to prevent or minimize mental health problems in pregnant women and optimize child development. In particular, our findings highlight the need to separate the concepts of stress and anxiety in future research and investigate the influence of timing of prenatal stress and/anxiety exposure on children's cognitive abilities. Finally, we argue that too little is known about the relationship between maternal prenatal stress and/or anxiety and children's domains of cognitive function, in particular in children older than 2 years old. It is evident that more studies are called to better understand this relationship and, in turn, optimize child cognitive development in a context where prenatal maternal stress and anxiety are commonplace.

## Supporting information


Appendix S1.
