# Supplementary material for: Association between prenatal maternal anxiety and/or stress and offspring's cognitive functioning: A meta‐analysis
Source: Child Dev. 2022 Dec 29;94(3):779–801. doi: 10.1111/cdev.13885 (PMC10952806; doi:10.1111/cdev.13885)
Supplement: Supplementary file 1 — Appendix S1. [file CDEV-94-779-s001.docx]

**Supplement tables**

**Table e1**

*Example of Search Strategy in Medline*

**Table e2**

*Key Demographic Features of the Studies Synthesised*

| **Studies** | **n** | **Sex (male, n)** | **Ethnicity^4^ (%)** | **Effect size** |
| --- | --- | --- | --- | --- |
| Bergman et al., 2007 | 123 | 48.8 | 83.7 | -0.41 |
| Buss et al., 2011 | 86 | 56.2 | 55.2 | -0.05 |
| Campbell et al., 2019 | 75 | 49.5 | 8.9* | 0.05 |
| Coplan et al., 2005 | 46 | n/a | 96.7* | -0.25 |
| Cortes Hidalgo et al., 2020 | 4,251 | 48.6 | 60.4 (Dutch) | -0.14 |
| D'Souza et al., 2019 | 5,768 | 51.4* | 68.8* (European) | -0.10 |
| DiPietro et al., 2006 | 82 | 40.0* | 85.1 | 0.15 |
| Gutteling 2006 | 112 | 44.6 | n/a | 0.00 |
| Huizink et al., 2002 | 170 | 49.4 | 96 | -0.11 |
| Jensen et al., 2014 | 6,964 | n/a | n/a | -0.03 |
| Keim et al., 2011 | 358 | 53.9 | n/a | -0.02 |
| Koutra et al., 2013 | 223 | 56.1 | 97.3 (Greek) | 0.04 |
| Koutra et al., 2017 | 288 | 55.6 | 95.5 (Greek) | 0.00 |
| Laplante et al., 2008 | 89 | 42 | 100 | -0.34 |
| Laplante et al., 2018 | 103 | 55 | 93.5* | -0.11 |
| Moss et al., 2017 | 145 | 56 | 96 | -0.01 |
| Nazzari et al., 2020 | 104 | 51 | 97.2 (Italian) | -0.18 |
| Pearson et al., 2016 | 3,270 | 50.3* | 96* | -0.04 |
| Plamondon et al., 2015 | 155 | 59 | 90 | 0.01 |
| Savory et al., 2020 | 76 | 46.1 | 96 | -0.19 |
| Simcock et al., 2017 | 115 | 47 | n/a | -0.11 |
| Simcock et al., 2019 | 104 | 52.7 | 97.6 | 0.05 |

Note. Based on IQ when available. ^4^ Caucasian, unless otherwise specified. ^5^ Average of 2yo and 4yo. * Based on the entire sample

**Table e3**

*Association Between Prenatal Stressors and Offspring Cognitive Outcomes*

| **Outcome** | **Stressors** | | |
| --- | --- | --- | --- |
|  | **Number of studies (sample size)** | **Correlations**  **r (95% CI)** | **I^2^ value, %** |
| **General intellectual skills** | 7 (542) | -0.12 (-0.31; 0.07) | 96.80 |
| **Attention** | 4 (7,127) | 0.004 (-0.02; 0.03) | 6.51 |
| **Language** | 3 (326) | -0.13 (-0.63; 0.37) | 78.34 |
